# Robotic Kinematic measures of the arm in chronic Stroke: part 1 – Motor Recovery patterns from tDCS preceding intensive training

**DOI:** 10.1186/s42234-021-00081-9

**Published:** 2021-12-29

**Authors:** Caio B. Moretti, Dylan J. Edwards, Taya Hamilton, Mar Cortes, Avrielle Rykman Peltz, Johanna L. Chang, Alexandre C. B. Delbem, Bruce T. Volpe, Hermano I. Krebs

**Affiliations:** 1 Department of Mechanical Engineering, Massachusetts Institute of Technology, 77 Massachusetts Avenue, office 3-137, Cambridge, MA 02139 USA; 2 Universidade de Sao Paulo, Avenida Trabalhador Saocarlense – 400, Sao Carlos, SP Brazil; 3 Moss Rehabilitation Research Institute, 60 Township Line Rd, Elkins Park, PA 19027 USA; 4 Icahn School of Medicine at Mount Sinai, 1 Gustave L. Levy Pl, New York, NY 10029 USA; 5 Rehabologym, 90 N Broadway, Irvington, NY 10533 USA; 6 Feinstein Institute for Medical Research, 350 Community Dr, Manhasset, NY 11030 USA

**Keywords:** Stroke, Kinematics, Outcome measures, Robotics, tDCS

## Abstract

**Background:**

Effectiveness of robotic therapy and transcranial direct current stimulation is conventionally assessed with clinical measures. Robotic metrics may be more objective and sensitive for measuring the efficacy of interventions on stroke survivor’s motor recovery. This study investigated if robotic metrics detect a difference in outcomes, not seen in clinical measures, in a study of transcranial direct current stimulation (tDCS) preceding robotic therapy. Impact of impairment severity on intervention response was also analyzed to explore optimization of outcomes by targeting patient sub-groups.

**Methods:**

This 2020 study analyzed data from a double-blind, sham-controlled, randomized multi-center trial conducted from 2012 to 2016, including a six-month follow-up. 82 volunteers with single chronic ischemic stroke and right hemiparesis received anodal tDCS or sham stimulation, prior to robotic therapy. Robotic therapy involved 1024 repetitions, alternating shoulder-elbow and wrist robots, for a total of 36 sessions. Shoulder-elbow and wrist kinematic and kinetic metrics were collected at admission, discharge, and follow-up.

**Results:**

No difference was detected between the tDCS or sham stimulation groups in the analysis of robotic shoulder-elbow or wrist metrics. Significant improvements in all metrics were found for the combined group analysis. Novel wrist data showed smoothness significantly improved (*P <* ·001) while submovement number trended down, overlap increased, and interpeak interval decreased. Post-hoc analysis showed only patients with severe impairment demonstrated a significant difference in kinematics, greater for patients receiving sham stimulation.

**Conclusions:**

Robotic data confirmed results of clinical measures, showing intensive robotic therapy is beneficial, but no additional gain from tDCS. Patients with severe impairment did not benefit from the combined intervention. Wrist submovement characteristics showed a delayed pattern of motor recovery compared to the shoulder-elbow, relevant to intensive intervention-related recovery of upper extremity function in chronic stroke.

**Trial registration:**

http://www.clinicaltrials.gov. Actual study start date September 2012. First registered on 15 November 2012. Retrospectively registered. Unique identifiers: NCT01726673 and NCT03562663.

**Supplementary Information:**

The online version contains supplementary material available at 10.1186/s42234-021-00081-9.

## Background

Every year 15 million people suffer a stroke globally, with two-thirds experiencing residual impairment (World Health Organization, 2012). Upper extremity (UE) hemiparesis is the most common impairment, with the prevalence as high as 80% in the acute and 40% in the chronic phase post-stroke (Lawrence et al., 2001). Patients often report dissatisfaction with their UE recovery and long-lasting, costly restrictions to participation in activities of daily living (ADLs) and quality of life (Lai et al., 2002). More effective, individualized, and targeted therapy interventions are vital to improve patient outcomes and address the demand for rehabilitation (Bernhardt et al., 2019; Lohse et al., 2014; Ovbiagele et al., 2013).

Robotic therapy for UE rehabilitation has shown promising results, but the benefits do not extend to all patients at all stages of their recovery (Lo et al., 2010; Milot et al., 2014; Mazzoleni et al., 2013; Hsieh et al., 2018). Several studies have attempted to augment the benefits of robotic therapy by combining interventions (Bolognini et al., 2011; Edwards et al., 2019). Edwards et al., 2019 investigated the effectiveness of combining intensive robotic therapy for the UE with anodal transcranial direct current stimulation (tDCS, Robot_tDCS_) compared to sham-tDCS (Robot_Sham_) in a chronic stroke population. Results showed 36 therapy sessions produced significant and clinically meaningful improvements in motor recovery (as measured by the Fugl-Meyer Assessment of Upper Extremity Motor Recovery after Stroke (FMA-UE)) of both the Robot_tDCS_ and Robot_Sham_ groups. However, the application of anodal tDCS did not confer further advantage as measured by clinical outcome measures (Edwards et al., 2019).

Combined therapy studies using tDCS have reported variability in effectiveness, even when adopting similar methodology (Edwards et al., 2019; Giacobbe et al., 2013; Lefebvre & Liew, 2017; Straudi et al., 2016). One reason for the lack of reproducibility could be the subjectivity and insensitivity of clinical outcome measures (Krebs et al., 2002; Bosecker et al., 2010; Semrau et al., 2013). Literature in this field has advocated using kinematics to study motor recovery post-stroke (Bernhardt et al., 2019; Semrau et al., 2013; Dipietro et al., 2012; Dukelow, 2017; Scott & Dukelow, 2011; Dukelow et al., 2012; Colombo et al., 2005; Reinkensmeyer et al., 2004). Studies to date suggest kinematics (collected during a robotic evaluation) can provide a standardized and objective measure of a patient’s motor control, correlating with well-known clinical measures(Bosecker et al., 2010; Semrau et al., 2013; Dipietro et al., 2012; Dukelow, 2017; Krebs et al., 2014; Agrafiotis et al., 2021), and have the potential to enhance research knowledge of treatment effects, clinical reasoning, and our understanding of stroke recovery (Bernhardt et al., 2019). Robotic evaluations allow numerous metrics to be generated from a single task and evaluation of both trained movements (to assess improvements within and between therapy sessions) as well as untrained movements (to assess generalization of training.) Robotic derived smoothness metrics, in particular, show good potential for quantifying motor recovery in patients post-stroke (Rohrer et al., 2002). There is no consensus on the best single approach to quantify smoothness, but most studies have built on the work by Flash and Hogan (1985), who define smoothness as a measure of jerk (Dipietro et al., 2012; Rohrer et al., 2002; Flash & Hogan, 1985). Subsequent studies explain changes in smoothness as a gradual blending of discrete submovements (Rohrer et al., 2002; Krebs et al., 1999), concluding that an accurate overview of changes in smoothness requires a concurrent analysis of submovement blending characteristics (Krebs et al., 1999). These kinematic micro-metrics are calculated in this study to provide a detailed and objective quantification of motor performance that may augment the findings of clinical outcome measures and our understanding of motor recovery.

Edwards and colleagues included, but did not report the results of, robotic evaluations which generated kinematic and kinetic data (Edwards et al., 2019). Here we explore if there was a difference in kinematic or kinetic measures of motor control between patients receiving Robot_tDCS_ and Robot_Sham_. A sub-analysis of kinematic and kinetic data investigates if the severity of motor impairment was a factor in the effectiveness of tDCS application, to determine if intervention outcomes can be optimized by targeting a sub-group of patients. Lastly we examine trends in the kinematic and kinetic data of the shoulder-elbow (by performing a combined group analysis) and the novel kinematic data of the wrist to investigate treatment effects of tDCS and robotics and further our understanding of UE motor recovery in the chronic post-stroke population.

## Methods

### Study overview

Subjects were enrolled from two sites, Burke Neurological Institute and Feinstein Institute for Medical Research, to participate in a double-blind, sham-controlled, repeated measures study. Candidates were eligible if they presented with a single ischemic stroke (> six months) and right hemiparesis. The robotic intervention involved 1024 movement repetitions per session, alternating shoulder-elbow (MIT-MANUS planar robot) and wrist-forearm robot therapy (MIT-WRIST, wrist robot) on separate days, performed three times a week (for a total of 36 sessions). Both the Robot_tDCS_ (anodal tDCS, 2 mA, affected hemisphere, M1/SO montage) and Robot_Sham_ interventions were administered at rest for 20 min immediately prior to each robotic session (Giacobbe et al., 2013). Baseline clinical and robotic evaluations were performed twice within 6 weeks prior to commencing the intervention (separated by at least one week to ensure a stable baseline), at completion of the 12-week intervention, and after six months post training. Details on the study design are presented in Edwards et al., 2019.

### Sample size calculation

This study’s sample size was based on the primary clinical outcome measure (FMA-UE) and not on kinematic or kinetic metrics (Edwards et al., 2019). According to (Lo et al., 2010) 12-weeks of UE robotic training was expected to result in a 2.88 point FMA-UE change in the chronic stage of rehabilitation. For a combined tDCS and robotic intervention, a FMA-UE change of 4.33 points was estimated. Using a two-sided alpha and a standard deviation of approximately 1.5, enrolling 56 subjects (28 per group) would result in 90% power to detect a difference of 1.45 in the FMA-UE between the intervention groups. An enrollment goal of 66 subjects (33 per group) was selected to allow for a 15% subject attrition rate.

### Kinematic and kinetic measures of the UE

Twenty kinematic macro and micro-metrics were derived from four shoulder-elbow and 19 macro and micro-metrics from three wrist evaluation tasks. Additional file 1 describes the evaluation tasks and the macro and micro-metrics generated for each evaluation, and Additional file 2 outlines the submovement (micro) metric definitions. Additional details about the shoulder-elbow robotic evaluations have been published previously (Bosecker et al., 2010; Krebs et al., 1999).

### Data analysis

#### Analysis of kinematic and kinetic data

A data analysis framework was developed (Moretti et al., 2020) to pre-process data sets generated by the robotic evaluations. This tool used formerly developed and tested calculations (Bosecker et al., 2010; Dipietro et al., 2007) to automatically process the robotic kinematic and kinetic measures of all study subjects.

The statistical analysis was designed to test the superiority of Robot_tDCS_ compared to Robot_Sham._ Statistical significance between Robot_tDCS_ and Robot_Sham_ was determined for each robotic measure (both macro and micro-metrics) by first conducting a Jarque-Bera test (α = 5%) to choose between a parametric or non-parametric test. As samples did not follow a normal distribution, the Wilcoxon-Mann-Whitney (one-tailed) test was used. A Bonferroni adjustment for repeated data measures (at admission, discharge, and follow-up) was performed, setting the significance level at *P <* .0167 for an α = .05 and three evaluations per subject.

For the overall combined analysis of both patient groups (Robot_tDCS_ and Robot_Sham_) from admission to discharge, we used the Wilcoxon signed rank (one-tailed) test. As before the results were deemed significant if *p* values were < .0167.

All statistical analyses were performed using MATLAB (Natick, MA, The Mathworks, Inc., vR2019b.)

#### Post-hoc exploratory analysis

The severity of patient’s UE impairment was defined by his/her baseline FMA-UE result. A brute force (exhaustive) search was conducted to establish the range of FMA-UE scores in which tDCS had a significant effect on the greatest number of robotic measures.

The brute force search first analyzed the largest window of FMA-UE scores (1 to 66, all study participants), identified Robot_tDCS_ from Robot_Sham_ subjects, and then applied the Wilcoxon-Mann-Whitney test for each robotic measure. Next the FMA-UE score window was reduced, from 1 to 65 then 2 to 66, and so on. The process of narrowing the window and offsetting the scale was carried out for every case, generating lists of *p*-values for each combination. The subgroup with significant p-values in the lowest quartile demonstrated the largest difference between Robot_tDCS_ and Robot_Sham_ outcomes.

### Missing data

**Fig. 1:**
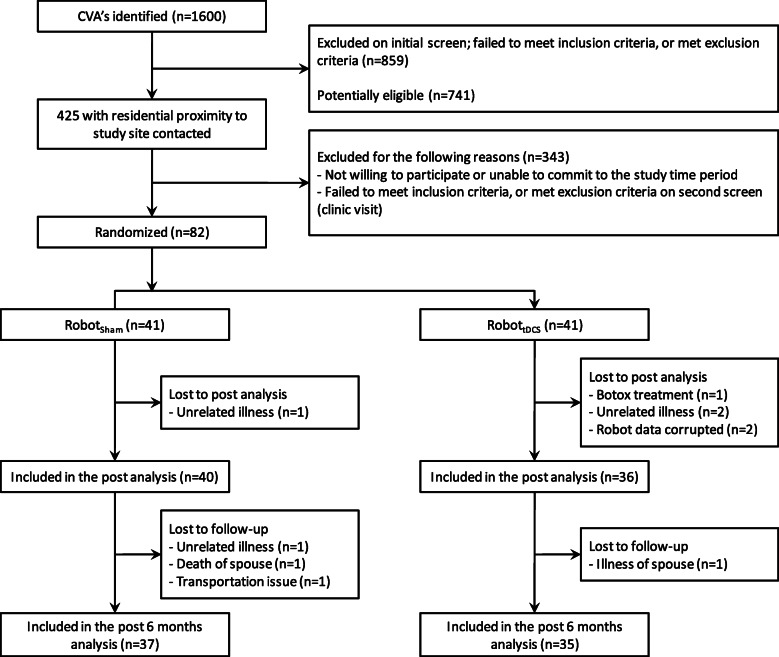
CONSORT flow diagram

Of the 82 subjects enrolled (Table 1), 76 were available for the discharge evaluation (an attrition rate of 7.32%, see Fig. 1). One subject in the Robot_Sham_ and two in the Robot_tDCS_ group experienced an unrelated illness, two subjects had corrupted robot data, and one subject was excluded when s/he underwent Botulinum Toxin A therapy. 72 subjects were included in the follow-up analysis due to unrelated illness of one subject, death/illness of a spouse, and transportation issues.

**Table 1:** Participant characteristics and clinical admission results of Robot_tDCS,_ Robot_Sham_, and the combined groups (overall)

Characteristic	Robot_**tDCS**_	Robot_**Sham**_	Overall
Participants (*n* [%])	41 [50.0%]	41 [50.0%]	82 [100.0%]
Age (mean years, [range])	66.4 [42–87]	69.2 [42–90]	67.8 [42–90]
Days from stroke to start of trial (mean days [range])	1475.2 [226–6935]	1160.0 [151–6936]	1317.6 [151–6936]
Gender (*n* Female [%])	16 [39.0%]	16 [39.0%]	32 [39.0%]
Stroke location (*n* cortical [%])	26 [63.4%]	27 [65.9%]	53 [64.6%]
FMA-UE (mean [range])	25.6 [7–57]	25.4 [7–55]	25.5 [7–57]
WMFT (mean [range])	60.0 [1–169]	56.0 [0–167]	58.0 [0–169]
BI (mean [range])	88.4 [10–100]	85.0 [15–100]	86.7 [10–100]
MRC (mean [range])	46.8 [8–79.5]	44.1 [15–85]	45.5 [8–85]

Note: *tDCS* transcranial direct current stimulation, *FMA-UE* Fugl-Meyer Assessment of Upper Extremity Motor Recovery after Stroke, *WMFT* Wolf Motor Function Test, *BI* Barthel Index, *MRC* Medical Research Council Motor Power score

Of note, there is a small discrepancy between the number of subjects included in the analysis reported in Edwards et al., 2019 and the number of subjects analyzed in the robotic metric analysis due to different evaluation methods being conducted during different sessions, by different assessors.

## Results

### Analysis of kinematic and kinetic data: effectiveness of tDCS

Completed robotic evaluation results were available for 76/82 enrolled subjects at discharge and 72 at six-month follow-up (Fig. 1). There was no noteworthy difference in any metric between the Robot_tDCS_ and Robot_Sham_ groups at admission, discharge, or follow-up for the shoulder-elbow or wrist data of all patients. Tables 2 and 3 show the between group analysis at the 3 evaluation time-points for the shoulder-elbow and wrist metrics found to be significant in the combined analysis (explained below and in Tables 4 and 5).

**Table 2:** Comparison of Robot_tDCS_ and Robot_Sham_ kinematic and kinetic metrics at admission, discharge, and follow-up for the shoulder-elbow

SHOULDER-ELBOW										
		**Admission**			**Discharge**			**Follow-up**		
**Robotic Task**	**Metric**	**Robot**_**tDCS**_ **mean (SD)**	**Robot**_**Sham**_ **mean (SD)**	**Group Comparison (*P*** **value)**	**Robot**_**tDCS**_ **mean (SD)**	**Robot**_**Sham**_ **mean (SD)**	**Group Comparison (*P*** **value)**	**Robot**_**tDCS**_ **mean (SD)**	**Robot**_**Sham**_ **mean (SD)**	**Group Comparison (*P*** **value)**
Unconstrained trained reaching (macro-metrics)	Deviation (m)	.030 (.032)	.030 (.028)	.511	.024 (.031)	.021 (.023)	.519	.027 (.033)	.022 (.023)	.437
	Mean Speed (m/s)	.084 (.050)	.081 (.034)	.540	.094 (.047)	.091 (.032)	.464	.103 (.086)	.093 (.031)	.372
	Speed shape	.487 (.067)	.500 (.079)	.218	.526 (.077)	.531 (.069)	.401	.526 (.076)	.532 (.064)	.322
	Jerk (m/s^3^)	112.927 (300.934)	76.191 (203.966)	.824	58.847 (156.297)	33.952 (22.507)	.639	85.173 (250.909)	35.645 (36.063)	.415
Unconstrained trained reaching (micro-metrics)	Sub-movement number	8.148 (3.774)	7.267 (3.850)	.080	6.995 (3.535)	6.811 (3.584)	.391	6.634 (2.819)	6.390 (2.831)	.298
	Sub-movement duration (s)	.556 (.170)	.0554 (.131)	.607	.589 (.138)	.622 (.114)	.143	.597 (.127)	.610 (.128)	.322
	Sub-movement overlap (s)	.266 (.080)	.261 (.066)	.763	.283 (.067)	.293 (.051)	.414	.289 (.057)	.294 (.060)	.326
Unconstrained untrained circle drawing	Circle ratio	.662 (.247)	.667 (.231)	.578	.738 (.241)	.767 (.190)	.410	.698 (.232)	.751 (.181)	.166
	Joint independence	.634 (.220)	.598 (.196)	.264	.551 (.198)	.494 (.151)	.168	.572 (.206)	.520 (.179)	.175
	Minor axis (m)	.105 (.050)	.103 (.044)	.611	.118 (.045)	.122 (.038)	.414	.110 (.046)	.117 (.038)	.212
Reaching against resistance	Maximum displacement (m)	.109 (.033)	.102 (.037)	.733	.114 (.032)	.113 (.030)	.647	.111 (.030)	.113 (.029)	.567
	Overall aim (radians)	.292 (.311)	.310 (.304)	.603	.244 (.313)	.213 (.274)	.230	.249 (.286)	.199 (.199)	.314
	Maximum displacement (m)	.109 (.033)	.102 (.037)	.733	.114 (.032)	.113 (.030)	.647	.111 (.030)	.113 (.029)	.567
Isometric stabilization	Scatter (m)	.023 (.010)	.021 (.010)	.190	.021 (.011)	.018 (.011)	.116	.020 (.011)	.018 (.011)	.178
	Offset (m)	.038 (.020)	.032 (.017)	.110	.034 (.020)	.028 (.015)	.171	.032 (.018)	.029 (.017)	.215
Shoulder-elbow kinetic evaluation	Shoulder strength (N)	33.353 (24.787)	34.182 (20.111)	.233	38.925 (27.531)	38.979 (23.207)	.326	37.613 (23.337)	38.325 (23.954)	.482

Note: * indicates significance at *p* < 0.0167

**Table 3:** Comparison of Robot_tDCS_ and Robot_Sham_ kinematic and kinetic metrics at admission, discharge, and follow-up for the wrist

WRIST										
		**Admission**			**Discharge**			**Follow-up**		
**Robotic Task**	**Metric**	**Robot**_**tDCS**_ **mean (SD)**	**Robot**_**Sham**_ **mean (SD)**	**Group Comparison (*P*** **value)**	**Robot**_**tDCS**_ **mean (SD)**	**Robot**_**Sham**_ **mean (SD)**	**Group Comparison** **(*P*** **value)**	**Robot**_**tDCS**_ **mean (SD)**	**Robot**_**Sham**_ **mean (SD)**	**Group Comparison (*P*** **value)**
Unconstrained trained pointing (macro-metrics)	Deviation (radians)	.165 (.121)	.147 (.136)	.312	.145 (.124)	.134 (.135)	.292	.148 (.112)	.134 (.132)	.328
	Speed shape	.290 (.060)	.305 (.061)	.240	.332 (.069)	.349 (.067)	.237	.343 (.070)	.339 (.064)	.731
Unconstrained trained pointing (micro-metrics)	Sub-movement number	9.308 (7.216)	8.103 (6.908)	.083	6.931 (3.547)	8.076 (5.350)	.744	7.891 (5.566)	7.479 (4.741)	.548
	Sub-movement overlap (s)	.175 (.138)	.182 (.126)	.346	.206 (.152)	.178 (.084)	.648	.217 (.179)	.179 (.112)	.735
	Sub-movement interpeak interval (s)	.251 (.151)	.260 (.189)	.333	.260 (.172)	.231 (.115)	.280	.266 (.189)	.225 (.118)	.269
Pointing against resistance	Maximum displacement (radians)	.176 (.083)	.179 (.081)	.525	.190 (.083)	.204 (.088)	.379	.176 (.077)	.197 (.072)	.072
Isometric stabilization	Offset (radians)	.152 (.090)	.129 (.234)	.269	.124 (.087)	.152 (.147)	.597	.138 (.097)	.106 (.221)	.447

Note: * indicates significance at *p* < 0.0167

**Table 4:** Significant changes from admission to discharge for the combined analysis of shoulder-elbow kinematic and kinetic data *

SHOULDER-ELBOW			
**Robotic Task**	**Metric**	**Admission to Discharge** **(raw result [95% CI])**	***P*** **value**
Unconstrained trained reaching macro-metrics	Deviation (m)	.007 [.004 to .010]	*<* ·001*
	Mean Speed (m/s)	.009 [.0002 to 0.019]	·03
	Speed shape	.035 [.022 to .048]	·001*
	Jerk (m/s^3^)	45.907 [6.811 to 99.157]	·006*
Unconstrained trained reaching micro-metrics	Submovement number	.804 [.033 to 1.595]	·02
	Submovement duration (s)	.050 [.024 to .078]	*<* ·001*
	Submovement overlap (s)	.024 [.010 to .040]	*<* ·001*
Unconstrained untrained circle drawing	Circle ratio	.085 [.056 to .114]	*<* ·001*
	Joint independence	.091 [.063 to .122]	*<* ·001*
	Minor axis (m)	.015 [.010 to .020]	*<* ·001*
Reaching against resistance	Maximum displacement (m)	.008 [.004 to .012]	*<* ·001*
	Overall aim (radians)	.068 [.026 to .110]	*<* ·001*
Isometric stabilization	Scatter (m)	.002 [−.00006 to .004]	*<* ·001*
	Offset (m)	.004 [.002 to .007]	*<* ·001*
S/E kinetic evaluation	Shoulder strength (N)	5.075 [2.080 to 8.092]	·001*

Note: *S/E* shoulder-elbow, * indicates significance at *p* < 0.0167

**Table 5:** Significant changes from admission to discharge for the combined analysis of wrist kinematic data

Wrist			
**Robotic Task**	**Metric**	**Admission to Discharge (raw result [95% CI])**	***P*** **value**
Unconstrained trained pointing macro-metrics	Deviation (radians)	.018 [.004 to .033]	·01*
	Speed shape	.043 [.029 to .059]	*<* ·001*
Unconstrained trained pointing micro-metrics	Submovement number	.625 [−.873 to 2.175]	.224
	Submovement overlap (s)	.013 [−.024 to .049]	.074
	Submovement interpeak interval (s)	.012 [−.033 to .057]	.411
Pointing against resistance	Maximum displacement (radians)	.018 [.00004 to .037]	·01*
Isometric stabilization	Offset (radians)	.002 [−.044 to .038]	·006*

Note: * indicates significance at *p* < 0.0167

### Analysis of kinematic and kinetic data: effectiveness of combined robotic and tDCS therapy

**Fig. 2:**
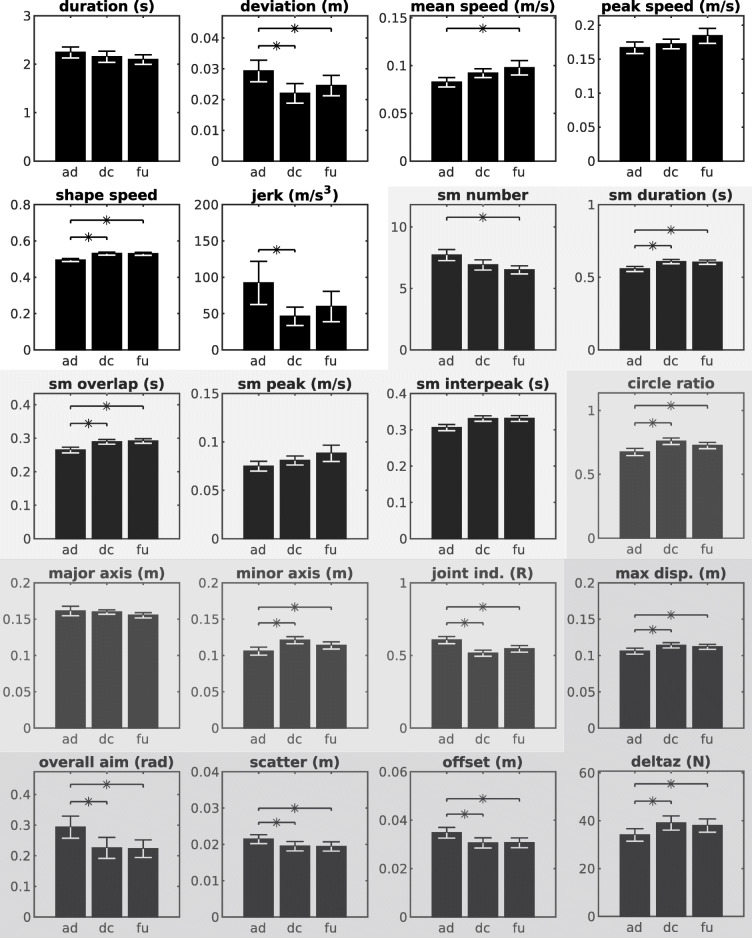
Combined Robot_tDCS_ and Robot_Sham_ mean and standard error of kinematic and kinetic outcome metrics for the shoulder-elbow (S/E.) Significant changes (*P <* .0167) between admission (ad), discharge (dc), and follow-up (fu.) are marked with an * Note: The bar-graphs in white background represent the unconstrained trained reaching macro-metrics, the lightest grey shading background represents the unconstrained trained reaching micro-metrics (sm= sub-movement), the slightly darker grey represents the unconstrained untrained circle metrics, and the darkest grey represents the reaching against resistance, isometric stabilization, and kinetic metrics (respectively, see Additional file 1 for further details on the metrics.)

**Fig. 3:**
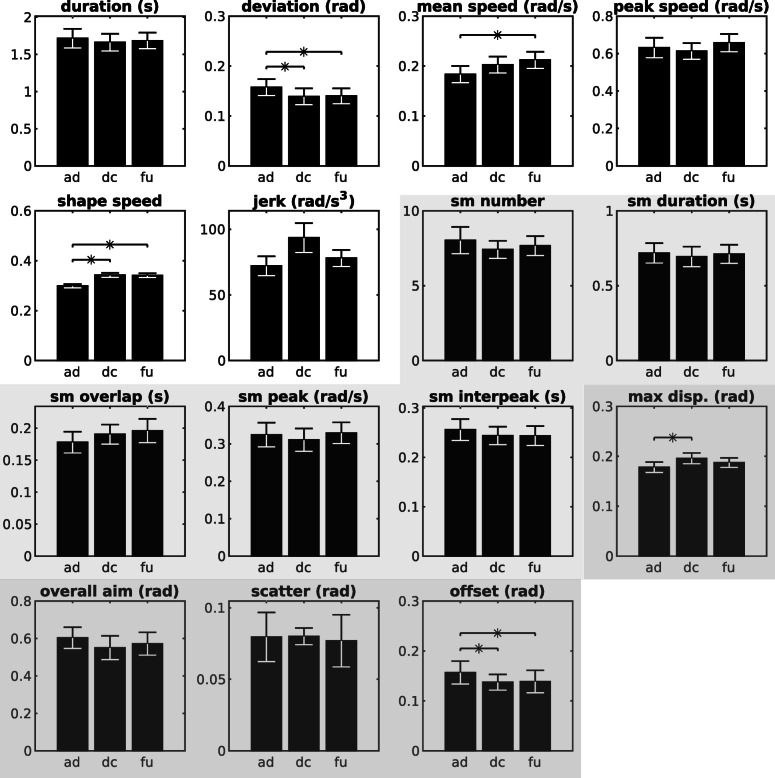
Combined Robot_tDCS_ and Robot_Sham_ mean and standard error of kinematic and kinetic outcome metrics for the wrist. Significant changes (*P <* .0167) between admission (ad), discharge (dc), and follow-up (fu.) are marked with an * Note: The bar-graphs in white background represent the unconstrained trained pointing macro-metrics, the lightest grey shading background represents the unconstrained trained pointing micro-metrics (sm= sub-movement), and the darkest grey represents the reaching against resistance, isometric stabilization, and kinetic metrics (respectively, see Additional file 1 for further details on the metrics.)

There were significant improvements in the kinematic and kinetic measures of motor control for the combined Robot_tDCS_ and Robot_Sham_ study population from admission to discharge (Tables 4 and 5, Figs. 2 and 3).

For the shoulder-elbow, 12/20 kinematic and kinetic metrics significantly improved and 2/20 were near significant (Table 4). For the unconstrained trained reaching task, these included: deviation, mean speed (trend only), speed shape, jerk normalized for terminated discrete movements (jerk), and submovement number (trend only), duration, and overlap (see Table 4, Additional file 1 and Additional file 2). Similarly, all metrics of the unconstrained untrained circle drawing task and both measures of the shoulder-elbow isometric stabilization task (scatter and offset) demonstrated a significant change. Both metrics of the reaching against resistance task (maximum displacement and overall aim) showed significant gains. The mean change in shoulder peak force (deltaz) improved significantly from admission to discharge.

For the unconstrained trained wrist pointing task, deviation and speed shape improved considerably. Submovement number trended down, overlap increased, and interpeak interval decreased, but there was no improvement in the measure of wrist jerk (− 21.456, − 46.063 to − 1.117; *P =* 0·89). Maximum displacement and offset improved for the wrist pointing against resistance and isometric stabilization task, respectively. All other metrics did not demonstrate a significant change (see Table 5 and Figs. 2 and 3.)

### Post-hoc analysis: stroke severity

For the shoulder-elbow, only patients with severe motor impairments (FMA-UE 7–9/66, *n* = 18) demonstrated a significant difference in kinematic metrics of motor control between groups, showing greater improvement for patients receiving Robot_Sham_ (*n* = 10) compared to Robot_tDCS_ (*n* = 8). Additional file 3 outlines the seven metrics that improved in the severe Robot_Sham_ sub-group (achieved significance or strong trend).

Similarly for the wrist, only patients with severe motor impairments (FMA-UE 8–12/66, *n* = 16) in the Robot_Sham_ group (*n* = 8) showed a significant difference in kinematic metrics (Additional file 3) from admission to follow-up. Remaining patient subgroups did not differ.

## Discussion

The comparison of Robot_tDCS_ and Robot_Sham_ outcomes using kinematic and kinetic metrics confirms the findings of the previously reported clinical outcomes (Edwards et al., 2019). Although significant motor gains were demonstrated between admission and discharge and admission and follow-up in both groups, the tDCS intervention was not shown to provide additional benefit at any time-point to the motor recovery of the UE than robotic therapy alone in the chronic stroke population.

This result is consistent with existing studies investigating combined robotic and tDCS therapy (Giacobbe et al., 2013; Straudi et al., 2016). Giacobbe et al., 2013 suggest tDCS may not have an additive or augmentation effect to the already significant motor gains induced by robotic therapy, but may instead change the nature of the training effect. It is possible that robotic training induces a ceiling effect whereby motor recovery is maximized, and any additional benefit from tDCS cannot be identified (Straudi et al., 2016). Comparing our study to other studies demonstrating either an additional positive effect of tDCS (Bolognini et al., 2011) or a difference in the kinematic measures of the tDCS group (Giacobbe et al., 2013) reveals that factors such as sample size, tDCS method, lesion site, motor task, stage of recovery, and type of stroke may contribute to the variance seen in patient outcomes (Lefebvre & Liew, 2017).

Combining the kinematic and kinetic results of the Robot_tDCS_ and Robot_Sham_ groups allowed us to investigate the value of these metrics as a measure of motor control and explore the trends in the UE motor recovery of the chronic stroke population. Edwards and colleagues reported a mean FMA-UE improvement of 7·36 points from baseline over the 12-week intervention (Edwards et al., 2019). The kinematic and kinetic measures showed a significant improvement from admission to discharge (Tables 4 and 5). Our findings support previous literature that a robotic evaluation and the metrics it generates are consistent with clinical measures and may provide a reliable, standardized, and objective tool for the quantification of motor recovery (Bosecker et al., 2010; Semrau et al., 2013; Dipietro et al., 2012) that may have the potential advantage of less interrater variability and subjectivity (Krebs et al., 2002).

The shoulder-elbow kinematic results were consistent with previous investigations, showing a progressive recovery in movement smoothness (speed shape, jerk, and submovement metrics), (Dipietro et al., 2012; Rohrer et al., 2004) shoulder strength, movement against resistance, (Bosecker et al., 2010) isometric stabilization, and coordination (circle tasks.) (Dipietro et al., 2007, Dipietro et al., 2012) and (Rohrer et al., 2004) used the same robotic evaluation to investigate the pattern of recovery of trained (unconstrained reaching) and untrained (circle drawing) movements in acute and chronic patients post-stroke by analyzing changes in smoothness and submovements. Both studies reported acute motor recovery of inpatients to be characterized by reduced movement duration, increased mean and peak speed, but a concurrent worsening of jerk. The submovement profile of the acute patients demonstrated fewer, larger movements being blended together (submovement number reduced and duration, interpeak interval, and overlap increased.) A similar pattern was evident for patients with chronic stroke, but smaller magnitude of change compared to acute; however, the jerk metric was seen to improve in the outpatient population. Our results replicate this pattern of chronic-stage stroke recovery, therefore supporting the value of both macro- and micro-kinematic measures to characterize motor recovery of the shoulder-elbow. Taken together, these findings support kinematic and kinetic metrics as sensitive and valid tools for assessing recovery of the UE during both the rapid and marked spontaneous recovery early after stroke, as well as intervention-related improvement during the stable recovery plateau.

**Fig. 4:**
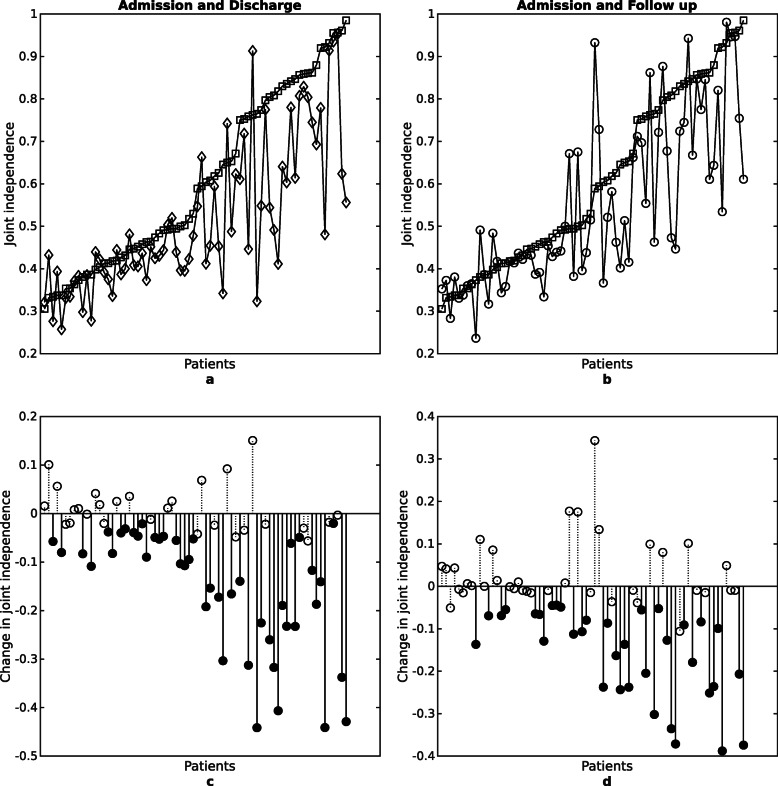
Improvements in joint independence over time. To afford direct comparison with Figs. 4 and 5 of (Dipietro et al., 2007), here we employed the same non-corrected significance at *P* < .05 level. Individual values of joint independence metric (**a** and **b** for all patients), sorted by their performance at admission (squares) in comparison to discharge (diamonds), and follow-up (circles). A lower number indicates greater/improving joint independence. Figures **c** and **d** represent changes in the joint independence metric, where filled circles indicate significance at discharge (**c**) and follow-up (**d**)

Improvements in the circle drawing task indicate that training point to point movements generalize motor gains to circle drawing and coordination of shoulder-elbow movements away from the body. Dipietro et al., 2012; Dipietro et al., 2007 also reported an increase in circle ratio and kinematic changes for the circle drawing task. Consistent with our results, Dipietro et al., 2007 reported improvements in the circle ratio predominately due to changes in the minor axes, not the major axes, of the circle drawing task. The minor axis corresponds primarily to independent elbow extension and shoulder abduction movements, suggesting that patients improve their ability to move out of the UE flexor synergy (elbow flexion and shoulder abduction) and gain greater joint independence (see Fig. 4 – to afford direct comparison with Dipietro we used the same significance level.) Circle drawing was not trained in this study or in Dipietro et al., 2012; Dipietro et al., 2007 and gains were sustained (six months after training,) indicating generalizability of training outcomes. This supports the use of motor learning as a better model, not adaptation (usually characterized by short-term changes), for motor recovery of the UE (Dipietro et al., 2012).

Wrist kinematic results generated from the combined analysis of all participants revealed a novel insight in motor recovery. In our chronic patient population, one measure of smoothness (speed shape) improved significantly but the jerk metric did not. A superficial interpretation suggests the intervention did not impact wrist smoothness. Nonetheless, wrist micro-metrics and submovement characteristics provide clarification on the nature of the smoothness changes. The number of submovements decreased, overlap increased, and there was a trend for reduced interpeak interval. These results indicate that submovements grew closer together in the chronic stage of recovery. Interestingly, this pattern of motor recovery replicates the shoulder-elbow submovement characteristics of the sub-acute inpatient population reported in Rohrer et al., 2004 and Dipietro et al., 2012. Although the chronic population within the same studies shared many trends in submovement characteristics, changes to interpeak interval appeared to be exclusive to the acute population for the shoulder-elbow. It is possible that both the proximal and distal segments of the UE share the same characteristics of recovery (as shown by a submovement analysis), but the time-course of the wrist recovery profile is relatively delayed compared to the shoulder-elbow. This conjecture may begin to explain findings in the literature such as the superior recovery of the wrist compared to the shoulder-elbow in chronic patients post-stroke. Hsieh et al., 2018 compared the effectiveness of distal versus proximal robotic therapy in patients six months or more after stroke onset. Results revealed participants receiving wrist robotic therapy had greater improvements in muscle strength and quality of movement in ADLs than patients receiving shoulder-elbow therapy (Hsieh et al., 2018). Although further studies are required to investigate this conjecture, our wrist submovement analysis suggests there may be potential for patients with distal impairment to continue to improve well into the chronic stage of their recovery. Our results attest to the importance and value of kinematic measures and a submovement analysis to enrich our understanding of UE motor recovery post-stroke.

This study is the first to our knowledge to investigate the impact of UE impairment severity on the effectiveness of tDCS. Most studies implementing tDCS recruit patients with a large range of impairment, (Bolognini et al., 2011; Giacobbe et al., 2013) despite neural recovery depending on the severity of stroke (Coupar et al., 2012; Kim & Winstein, 2017). Our results revealed the only patient subgroup with a significant difference in kinematic measures of motor control between Robot_tDCS_ and Robot_Sham_ had very severe motor impairment in favor of the Robot_Sham_ group. This result is interesting for two reasons.

Firstly, most studies investigating stroke prognosis have concluded that patients with less burden of disease (measured by the functional integrity of the corticospinal tract or the structural integrity of descending white matter pathways) have better functional outcomes (Coupar et al., 2012; Kim & Winstein, 2017). Nonetheless. other studies have also reported more significant improvements in the chronic patient population or those with lower levels of cortical activity and greater UE impairment. Straudi et al., 2016 showed significant and positive therapy outcomes for a combined tDCS and robotics intervention that was dependent on stage of recovery (patients with chronic stroke improved more than the acute group) and lesion location (subcortical was superior to cortical). Milot et al., 2014 investigated predictors of functional gain for patients receiving robotic therapy and found that lower baseline motor evoked potential (MEP) activation correlated with better functional outcomes of the affected UE. The explanation offered for these findings was: the lower baseline cortical activity may represent underused cortical potential, therefore patients with higher cortical activation and baseline measures of motor control may have less reserve to augment either cortical activity or behavioral changes with therapy (Milot et al., 2014; Straudi et al., 2016).

Secondly, the Robot_Sham_ group were shown to have better kinematic outcomes than Robot_tDCS_ in the severe subgroup of patients. Edwards also reported significant clinical findings of improved FMA-UE response rate (MCID) greater in the Robot_Sham_ group (Edwards et al., 2019). The mechanisms for this result are unknown, although we speculate that increases in abnormal tone could have been a factor. Data on tone was not collected in this study to inform this theory, but we believe this warrants further investigation. As a difference between groups was only observed in patients with very severe UE impairments, this speaks to the diversity of interactions of tDCS between different deficits, cortical activity levels, and individual patients (Krause & Kadosh, 2014).

## Conclusions

Significant improvements in motor control were detected with kinematic and kinetic measures for a combined UE robot and tDCS intervention in chronic stroke. However, no difference was found between the Robot_Sham_ and Robot_tDCS_ groups. This confirmed the results of previous clinical measures (Edwards et al., 2019). Submovement characteristics provided insight into improvements in smoothness and highlighted the importance of kinematic micrometrics for enhancing our understanding of motor recovery post-stroke. Novel wrist kinematic data suggested an interesting pattern of delayed wrist motor recovery in the chronic stage compared to the timeline of the shoulder-elbow. Our subgroup analysis revealed that patients with very severe motor impairment in the Robot_Sham_ group benefited the most from the intervention. Our study demonstrated limited benefit for adding anodal tDCS prior to robotic training in chronic stroke and future studies are needed to investigate the interaction of intensive motor training with other forms of neuro-stimulation and patient populations.

## Supplementary Information

**Additional file 1.** Outline of robotic evaluation tasks and the metrics derived from the evaluations ^a^

**Additional file 2.** Description of submovement (micro) metrics ^a^

**Additional file 3.** Post-hoc exploratory analysis results: significant changes or strong trends seen in the Robot_sham_ group in the severe patient sub-group ^a^

## Data Availability

We anticipate that the data captured and created by this research will be of broad interest to communities engaged in research on human motor behavior. Data generated by this research project will be made publicly accessible through a portal linked to our lab homepage http://the77lab.mit.edu/. Access to these data will be “read-only” and password protected. Passwords will be made freely available upon submission of a request by email agreement that the source of the data will be acknowledged in any publication arising from use of these data.

## Abbreviations

tDCS: Transcranial direct current stimulation
UE: Upper extremity
ADLs: Activities of daily living
FMA-UE: Fugl-Meyer Assessment of Upper Extremity Motor Recovery after Stroke
