# Supplementary material for: Robotic Kinematic measures of the arm in chronic Stroke: part 1 – Motor Recovery patterns from tDCS preceding intensive training
Source: Bioelectron Med. 2021 Dec 29;7:20. doi: 10.1186/s42234-021-00081-9 (PMC8715636; doi:10.1186/s42234-021-00081-9)
Supplement: Supplementary file 1 — Additional file 1. Outline of robotic evaluation tasks and the metrics derived from the evaluations a [file 42234_2021_81_MOESM1_ESM.docx]

**Additional file 1: Outline of robotic evaluation tasks and the metrics derived from the evaluations ^a^**

| **Robotic Evaluation** | **Description** | **Macro and Micro-Metrics Derived** |
| --- | --- | --- |
| Unconstrained reaching task (S/E robot) | Required the patient to attempt 80 active reaching motions to and from eight targets spaced equally around a 14cm circle. The reaching movements used for the evaluation were similar to the robot assisted tasks completed during training.[17] | Macrometrics=Duration, deviation, mean speed, peak speed, speed shape (ratio of mean to peak speed), and jerk (normalized for terminated reaching movements.) Micrometric data was derived by extracting support-bounded lognormal submovements from movement speed profiles as described in Rohrer et al.[27] This included submovement number, duration, overlap, peak, and interpeak interval (see Additional file 2 for submovement definitions.) |
| Unconstrained wrist pointing task | Required the patient to attempt 80 active wrist (F/E/RD/UD) motions to and from 8 targets distributed around an ellipse with major axis of 60^o^ (30^o^ for F/E each) and minor axis of 30^o^ (15^o^ for RD/UD each). The wrist pointing movements used for the evaluation were similar to the robot assisted tasks completed during training.[17] | Macrometrics=Duration, deviation, mean speed, peak speed, speed shape (ratio of mean to peak speed), and jerk (normalized for terminated pointing movements.) Micrometrics=submovement number, duration, overlap, peak, and interpeak interval (see Additional file 2 for submovement definitions.) |
| Unconstrained circle drawing task (S/E robot only) | Involved the patient completing 5 unassisted attempts to draw a circle, in a clockwise and counterclockwise direction, from 2 different starting positions (3 o’clock and 9 o’clock) for a total of 20 movement repetitions. Note that training did not include attempts to draw circles. | Macrometrics=Major and minor axes of the best-fitting ellipse and the ratio of the axes measurements for each of the 4 circle drawing conditions as well as the orientation of the major axes. Inverse kinematics allow us to estimate the shoulder and elbow joint movements. Joint independence determines the correlation between the shoulder and elbow movement. |
| Movement against resistance task | Required the patient to move against an increasing force as they reach toward the targets. | Macrometrics= maximum displacement and overall aim. |
| Isometric stabilization task | The patient attempted to hold their S/E or wrist still while the robot exerted forces to move the patient’s arm/robot handle toward the outer edge of the circle. | Macrometrics=Movement scatter and offset. |
| Kinetic S/E evaluation (S/E robot only) | The patient was positioned facing the robot (for shoulder F/E) or rotated 90 degrees away from the robot (for shoulder AB/AD) in 90 degrees of shoulder F, with the elbow fully extended and the forearm, wrist, and hand supported by the robot arm. The patient was asked to attempt to lift their arm (for F and AB measurements) or push down (for E and AD measurements) five times in each direction, for a total of 20 trials. | Macrometrics=Mean shoulder strength (deltaz) |

^a^ S/E= shoulder-elbow, F= flexion, E=extension, AB= abduction, AD= adduction, RD= radial deviation, UD= ulnar deviation
