# Supplementary material for: Robotic Kinematic measures of the arm in chronic Stroke: part 1 – Motor Recovery patterns from tDCS preceding intensive training
Source: Bioelectron Med. 2021 Dec 29;7:20. doi: 10.1186/s42234-021-00081-9 (PMC8715636; doi:10.1186/s42234-021-00081-9)
Supplement: Supplementary file 2 — Additional file 2. Description of submovement (micro) metrics a [file 42234_2021_81_MOESM2_ESM.docx]

**Additional file 2: Description of submovement (micro) metrics ^a^**

| **Submovement Metric** | **Definition ^b^** |
| --- | --- |
| Number | The number of submovements in an entire movement |
| Duration (s) | The time from initiation until the termination of an individual submovement |
| Overlap (s) | Interval between commencement of a submovement and termination of the previous submovement |
| Peak  (m/s (S/E)^a^  rad/s (wrist)) | Peak speed of each individual submovement |
| Interpeak interval (s) | Interval between peaks of consecutive submovements |

^a^ S/E= shoulder-elbow.

^b^ Metric definitions were adapted from Rohrer et al.[27]
