# Supplementary material for: Robotic Kinematic measures of the arm in chronic Stroke: part 1 – Motor Recovery patterns from tDCS preceding intensive training
Source: Bioelectron Med. 2021 Dec 29;7:20. doi: 10.1186/s42234-021-00081-9 (PMC8715636; doi:10.1186/s42234-021-00081-9)
Supplement: Supplementary file 3 — Additional file 3. Post-hoc exploratory analysis results: significant changes or strong trends seen in the Robotsham group in the severe patient sub-group a [file 42234_2021_81_MOESM3_ESM.docx]

**Additional file 3: Post-hoc exploratory analysis results: significant changes or strong trends seen in the Robot_sham_ group in the severe patient sub-group ^a^**

| **Shoulder-elbow tasks** | | | | | | | |
| --- | --- | --- | --- | --- | --- | --- | --- |
|  | **Metric** | **Admission to Discharge (raw result [95% CI])** | ***P* value** | **Admission to follow-up (raw result [95% CI])** | ***P* value** | **Discharge to follow-up (raw result [95% CI])** | ***P* value** |
| Unconstrained reaching | Submovement duration (s) | .165  [.027 to .296] | .03 |  |  | 0.154  [.009 to .291] | .02 |
|  | Submovement overlap (s) | .081  [.014 to .149] | .03 |  |  |  |  |
|  | Jerk (m/s^3^) |  |  | 226.26  [-15.015 to 587.575] | ·02 |  |  |
| Movement against resistance | Overall aim (radians) |  |  | .252  [.039 to .476] | ·02 |  |  |
| Circle drawing | Circle ratio | .125  [.030 to .226] | ·03 | .197  [.109 to .290] | *<* ·001 |  |  |
|  | Joint independence | .141  [.040 to .240] | .01 |  |  |  |  |
|  | Minor axis (m) | 035  [.014 to .057] | ·004 | .043  [.025 to .063] | ·001 |  |  |
| **Wrist tasks** | | | | | | | |
|  | **Metric** | **Admission to Discharge (raw result [95% CI])** | ***P* value** | **Admission to follow-up (raw result [95% CI])** | ***P* value** | **Discharge to follow-up (raw result [95% CI])** | ***P* value** |
| Unconstrained pointing | Submovement interpeak interval (s) |  |  | .100  [.032 to .200] | ·007 |  |  |
| Movement against resistance | Overall aim (radians) |  |  | 0.288  [.013 to .554] | ·01 |  |  |

^a^ Empty fields indicate no significant change in the metric at that timepoint
